# A Methodology to Systematically Investigate the Diffusion Degradation of Cemented Carbide during Machining of a Titanium Alloy

**DOI:** 10.3390/ma12142271

**Published:** 2019-07-15

**Authors:** Sara Saketi, Jonas Östby, Ulf Bexell, Mikael Olsson

**Affiliations:** 1 Material Technique, Dalarna University, SE-791 88 Falun, Sweden; 2 Material Technique, Uppsala University, SE-581 83 Uppsala, Sweden; 3 R&D, AB Sandvik Coromant, SE-683 85 Sandviken, Sweden

**Keywords:** turning, Ti6Al4V, wear, tool, chip, SEM, AES, ToF-SIMS

## Abstract

Using Ti6Al4V as a work material, a methodology to systematically investigate the diffusion degradation of cemented carbide during machining is proposed. The methodology includes surface characterization of as-tested worn inserts, wet etched worn inserts, metallographic cross-sectioned worn inserts as well as the back-side of the produced chips. Characterization techniques used include scanning electron microscopy (SEM), energy dispersive X-ray spectroscopy (EDS), Auger electron spectroscopy (AES) and time of flight secondary ion mass spectroscopy (ToF-SIMS). The results show that the characterization of wet etched worn inserts gives quick and useful information regarding the diffusion degradation of cemented carbide, in the present work the formation of a fine crystalline W layer (carbon depleted WC layer) at the tool-work material interface. The present study also illuminates the potential of AES analysis when it comes to analyzing the degradation of cemented carbide in contact with the work material during machining. The high surface sensitivity in combination with high lateral resolution makes it possible to analyze the worn cemented carbide surface on a sub-µm level. Especially AES sputter depth profiling, resulting in detailed information of variations in chemical composition across interfaces, is a powerful tool when it comes to understanding diffusion wear. Finally, the present work illustrates the importance of analyzing not only the worn tool but also the produced chips. An accurate characterization of the back-side of the chips will give important information regarding the wear mechanisms taking place at the tool rake face–chip interface. Surface analysis techniques such as AES and ToF-SIMS are well suited for this type of surface characterization.

## 1. Introduction

Today, the most common high production tool materials in the manufacturing industry are coated and uncoated cemented carbides. A comprehensive overview of cemented carbides in use by the machining industry has recently been published by García et al. [1], highlighting the possibilities of tailoring the chemical compositions and microstructures of tools and inserts to meet the often severe thermal and mechanical conditions in different machining applications. However, designing a material to avoid premature tool failure (fracture), increase tool life and reduce the wear can require detailed knowledge of the mechanisms underlying the wear on microscales.

Cutting tool wear is the result of the important tribosystem and involves many complex chemical, physical and thermo-mechanical phenomena resulting in different wear mechanisms, e.g., abrasive wear, adhesive wear, chemical wear and diffusion wear [2,3].

The general trend to higher productivity through higher cutting speeds has illuminated the importance of studying the mechanisms responsible for cutting tool wear at high cutting speeds, i.e., when chemical and diffusion wear dominate [4,5] due to high temperature. Temperatures for cutting speeds up to 120 m/min are estimated to be well over 1000 °C by Su et al. [4], and it has been established by Jawaid et al. [5] and Satyanarayana et al. [6] that cutting speed is the machining parameter which most significantly correlates to cutting zone temperature during Ti-alloy machining. Understanding the mechanisms underlying chemical interactions between cutting tool and adhered material is useful for the design of new cemented carbide grades, coatings and cutting tool life estimations.

Titanium and its alloys are challenging to the manufacturers due to typical combinations of the thermal, chemical and mechanical properties and the chip formation characteristics. Titanium alloys are materials with comparatively high temperature strength and pronounced work ability [7]. Some well-established challenges in machining these materials are the segmented nature of the generated chips [8], the low module of elasticity [4] the small chip/tool contact length [9] the low thermal conductivity [10] and the high chemical reactivity [11,12,13,14]. Although a relatively large number of surveys have been performed focusing on the diffusion controlled degradation of cemented carbide tools, the detailed picture is far from complete and there is still a lack of high quality studies focusing on the mechanisms being active on a microstructural level at the chip-tool rake face interface using techniques such as Auger electron spectroscopy (AES), time of flight secondary ion mass spectroscopy (ToF-SIMS) and transmission electron microscopy (TEM).

Diffusion wear in titanium alloy machining was studied by Ramirez et al. [15], noting that diffusion wear is an important issue but is scantily understood. Diffusion couples were used, and it was found that a TiC layer was formed, but electron probe microanalysis (EPMA) could not confirm this on tools used in machining experiments, which was presumed to be due to the small amounts of TiC present on the tool due to continuous wear. Still, it was proposed that the TiC layer rate-limited wear by blocking diffusion of W. The presence of a TiC layer was also found in a recent crossed-cylinders experiment performed by Olander and Heinrichs [16] in which the growth of the layer was also investigated. The wear of the cemented carbide was found to have several stages where Co binder was first worn away, after which TiC was formed by adhered material. The amount of TiC was however greatly reduced in the interface as the underlying WC was carbon depleted, suggesting that this depletion rate-limits the wear. It can be noted that the crossed-cylinders experiments were performed by sliding two surfaces against each other, which involves the presence of oxides that would not normally be present in the center of a wear zone during machining.

Hartung and Kramer [17] performed AES surface analysis of a WC-Co cemented carbide insert used for turning in Ti-6Al-4V for 30 s and found the indication of a TiC layer in the worn crater. However, no observations about layer thicknesses are given. Min and Youzhen [18] and Ikuta et al. [19] performed AES point analysis and found the presence of a reaction phase of Ti and C which may form at the cemented carbide–Ti-alloy interface during the cutting process. Nouari and Makich [20] performed line-scan AES analysis of a polarized cross section of a WC-Co insert with the intention to analyze the diffusion zone between adhered Ti-6Al-4V and cemented carbide. They observed diffusion of elements from the machined material (Ti, Al, and V) to the cemented carbide cutting tool (W, Co, and C) and vice versa. However, the work provides no distinction between the signals of C bonded to W and of C bonded to Ti, which prevents any detailed evaluation of layer thicknesses or element concentration gradients.

Jawaid et al. [21], using EDS, found the evidence of diffusion of Co and W into the work material both at the flank and rake faces of the cutting tool when face milling Ti6Al4V. Jianxin et al. [22], using EDS, found evidence of diffusion of W and Co of the cemented carbide to the Ti6Al4V alloy, and Ti, Al and V of the Ti6Al4V alloy to the cemented carbide in the crater area. Zhang et al. [23] proposed that the pulling out and removing of the WC particles due to diffusion of cobalt into the Ti6Al4V chip dominated the crater wear mechanism. By using EDS, they observed the diffusion of W and Co into the adhered work material and Ti into the cemented carbide. Liang et al. [24] found diffusion of W and Co to adhering Ti6AlV and diffusion of Ti into cemented carbide through line scan EDS of a cross section of WC-CO. Rahman Rashid et al. [25] using EDS, proposed a number of concurrent processes for crater wear of cemented carbide during Ti6Al4V-machining, including the diffusion of C, W and Co into the diffusion interface. However, none of the above studies gives information regarding the diffusion depths and thickness of the diffusion zones. For EDS, the relative large information depth and poor lateral resolution (especially when using a high primary electron energy) also limits the resolution of the element identification, making the above results questionable. Recently, Odelros et al. [26] and Kaplan et al. [27] using SEM, EDS and X-ray diffraction (XRD), found evidence of carbon depletion of the WC resulting in the formation of W layer and (Ti,V)C at the cemented carbide–Ti6Al4V interface in turning. Also, Latteman et al. [28], using SEM and scanning transmission electron microscope (STEM) in combination with EDS and electron energy loss spectroscopy (EELS), found the formation of a W layer and a Ti and Co diffusion zone at the interface. The work by Odelros et al. [26] additionally modeled the diffusion of Ti in the binder of cemented carbide. Efforts to model diffusion wear are also being made in a work by Edin et al. [29], using a mechanism based on carbon depletion of WC in contact with Ti.

Measurements of the materials and element concentration gradients that are relevant for verification or modeling of a proposed wear mechanism require high precision. Although formation of a titanium carbide layer, which is found in diffusion couples, can also be verified using AES analyses of worn tools, it is not yet clarified if the method can investigate thicknesses and element gradients in the wear zone. Static diffusion couple tests can provide fundamental information about the material transfer in an interface at a particular temperature and pressure, but they do not necessarily represent the intensely dynamic conditions of a machining operation. For example, a cutting zone contact could in some scenarios be better represented by a dissolution of elements from the cutting tool into an empty reservoir, which is continuously renewed, with no saturation of the diffusing elements at the tool–work material interface. To model the diffusion in an interface in a way similar to, e.g., Odelros et al. [26] and then compare results to an actual wear interface, all available data on element concentration gradients in the wear contact could be considered valuable.

In the present study, a methodology to systematically investigate the diffusion degradation of cemented carbide during machining is proposed using Ti6Al4V as a work material. The methodology includes surface characterization of as-tested worn inserts, wet etched inserts, metallographic cross-sectioned as-tested worn inserts as well as back sides of the chips that have been produced. Characterization techniques used include AES, ToF-SIMS as well as SEM. Results are discussed with respect to the information obtained and the pros and cons associated with the different approaches used.

## 2. Experimental

### 2.1. Materials

**Figure 1:**
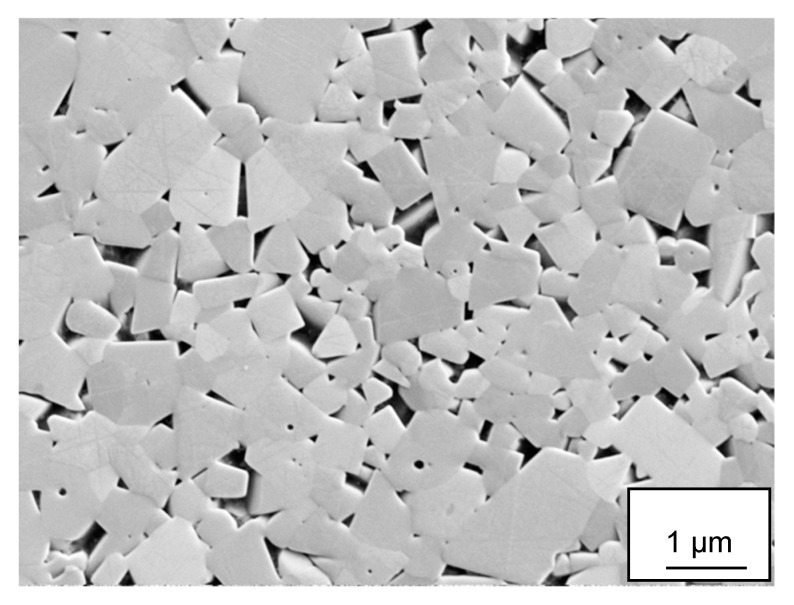
Microstructure of the cemented carbide grade investigated. WC phase appears bright, Co phase appears dark.

The cemented carbide grade used in the present study, H13A (Sandvik Coromant, Sandviken, Sweden), is a commercially available uncoated WC-Co grade which combines toughness and good wear resistance for the turning of heat resistant steels and titanium alloys at moderate cutting speeds and feeds. Figure 1 shows the microstructure of the cemented carbide grade while Table 1 shows hardness and chemical composition. The work material used in the present study was a mill-annealed bar with diameter of 180 mm of Ti6Al4V alloy, grade 5, supplied by Timet (Dallas, TX, USA) and then it was machined down 1 mm (so 2 mm in total) to remove any skin to get a smooth surface (see Table 2).

### 2.2. Turning Tests

**Figure 2:**
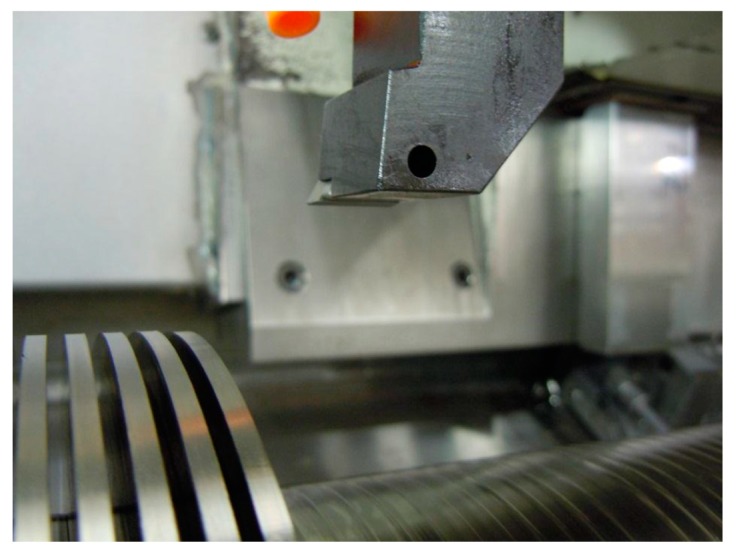
Test set-up for orthogonal turning tests using a flanged Ti6Al4V work piece.

Flat-faced TCMW 16T304 WC-Co inserts were used in orthogonal turning tests in a George Fischer CNC lathe, NDM-17/125. The cutting tool holder used was an STFCR2525M 16. Flat-faced TCMW 16T304 WC-Co inserts were used in orthogonal turning tests in a George Fischer CNC lathe, NDM-17/125. The cutting tool holder used was an STFCR2525M 16. For the orthogonal cutting approach, a radial turning operation was performed on workpiece that was pre-prepared with flanges of 30 mm depth that were 3 mm wide and 3 mm apart (see Figure 2). Based on the established importance of cutting velocity on temperature, machining parameters were selected in a way that would correspond to relatively low temperatures (test series I) and relatively high temperatures (test series II) at the tool rake face-chip interface. In this context, the lower temperature should be one where no or very little reactant is formed by a reaction causing wear in the cutting zone. Since specific details of the wear mechanism can be presumed to be unknown, the cutting speeds were chosen to cause significant (high speed) and insignificant (low speed) wear based on previous knowledge [30]. This was achieved by the following combinations of cutting parameters:

Test series I: cutting speed *v_c_*: 30 m/min, feed rate *f_n_*: 0.2 mm/rev, cutting depth *a_p_*: 3.0 mm.
Test series II: cutting speed *v_c_*: 90 m/min, feed rate *f_n_*: 0.2 mm/rev, cutting depth *a_p_*: 3.0 mm.

All cutting tests were run for 30 s and performed with a 6% solution of a semi synthetic coolant, Hocut B50S, with a pH of 9.5 and a maximum flow rate of 200 L/min.

For both test series, separate cutting edges were run for 0.5, 1, 2 and 3 min, respectively. The test series were duplicated. At this point, it can be noted that no significant differences over time were observed for the volumetric wear rate within either test series, but the results regarding wear rate are presented in another paper [30].

### 2.3. Post-Test Evaluation

#### 2.3.1. Sample Preparation

Besides the characterization of the as-tested cemented carbide inserts, two different approaches were used for the post-test characterization;

(i) Etching the inserts in 40% hydrofluoric acid for 15 min at room temperature to remove the adhered work material from the cutting zone, thus exposing the underlying worn cemented carbide.

(ii) Preparation of cross-sections perpendicular to cutting edge in the center of the worn crater on the rake face. The cross-sections were prepared by conventional metallographic techniques, including precision cutting using a diamond disc, grinding and polishing using a standard colloidal silica suspension (0.04 µm) in the last step.

#### 2.3.2. Characterization of Worn Tool Surfaces and Back-Side of Chips

The worn rake faces of the cutting inserts and the back-sides of the produced chips were characterized using high resolution scanning electron microscopy (FEG-SEM, Zeiss, Oberkochen, Germany, Ultra 55) in combination with energy dispersive X-ray spectroscopy (EDS, Oxford Inca Energy, Abingdon, UK). For the SEM and EDS analyses, interaction was limited while increasing sensitivity to light elements through use of low primary electron energies in the typical range 3–5 keV (resulting from using an acceleration voltage of 3–5 kV).

The top surfaces of worn tools and the back sides of chips were analyzed using the two very surface-sensitive methods AES and ToF-SIMS. These experiments were performed to evaluate any presence of diffusion zones or tribo chemical layers.

An Ulvac-PHI 700 Xi Scanning Auger Nano probe (Ulvac-PHI, Chigasaki, Japan) was used to perform AES analyses of the diffusion of specific elements of tool material into the work material. In these analyses, the accelerating voltage was 10 kV and the primary beam current was 10 nA. For depth profiling, 2.0 kV Ar^+^ ion sputtering with a sputter rate of 17.1 nm/min was used. The sputter rate was measured on a Ta_2_O_5_ reference sample with 100 nm thickness. The Auger depth profile data were evaluated with software from PHI-Matlab (version 9.3). Different chemical states of the identified elements were extracted by use of linear least squares (LLS) fitting.

ToF-SIMS analysis (PHI TRIFT II) was performed with a pulsed liquid metal ion gun (LMIG) with a source enriched in ^69^Ga isotopes. To obtain depth profiles, a surface of 100 μm × 100 μm was sputtered, using a continuous non-pulsed beam with primary ion energy of 15 keV and an aperture giving a current of ~600 pA. Furthermore, a 50 μm × 50 μm surface in the centre of the sputtered region was analyzed using positive static SIMS mode. In calibrations of all SIMS spectra, peaks with known mass/charge ratios were used.

**Figure 3:**
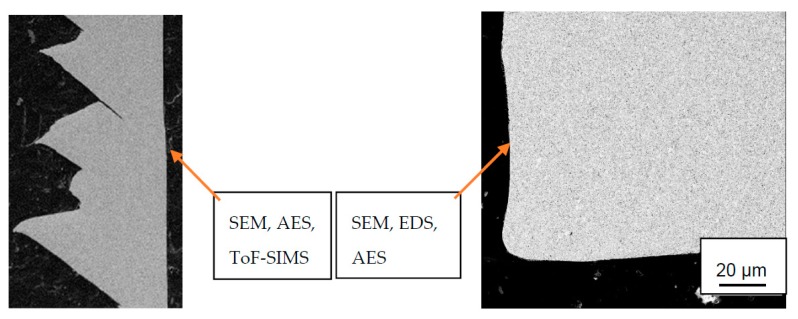
Cross-sections of Ti6Al4V chip and cemented carbide insert (to scale) and applied surface analysis techniques in this study.

These experiments were repeated 3 times at different locations in each wear scar. Figure 3 illustrates the chip and cutting zone for an orthogonal cutting process after 0.5 min cutting time with cutting parameters *v_c_* = 115 m/min, *f_n_* = 0.2 mm/rev, *a_p_* = 3 mm and the surface analysis techniques which are applied.

## 3. Results

Both test series showed some degree of adhesion of workpiece material. However, for test series I, no indication of chemical interaction under the adhesion layer was found. For test series II, there were clear indications of chemical interaction under the layer, but no significant differences in the reaction products were found for the different cutting times. The results that are presented here are representative for all measurements.

### 3.1. SEM and EDS Analysis of Worn Inserts

**Figure 4:**
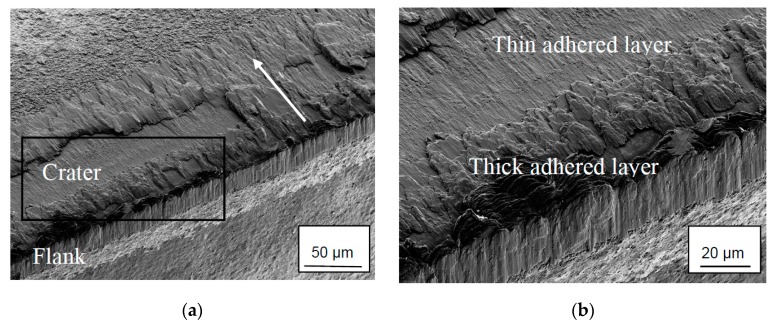
(**a**) Wear characteristics of cemented carbide insert showing the crater and flank wear regions. Cutting parameters: *v_c_*: 90 m/min, *f_n_*: 0.2 mm, *t*: 0.5 min. The arrow shows the chip flow direction. Note the transfer of work material to the crater and flank wear regions and especially to the cutting edge and close to the depth-of-cut. (**b**) Detail of (**a**) showing transferred work material covering the worn cemented carbide.

Figure 4 shows a worn cemented carbide cutting insert after 0.5 min turning in the Ti6Al4V alloy using a high cutting speed (*v_c_*: 90 m/min). As can be seen, the worn crater and flank wear regions show extensive transfer of work material welded to the tool surface making it extremely difficult to characterize the worn cemented carbide underneath the adhered work material. It should be noted that the same tendency was observed also at the low cutting speed (*v_c_*: 30 m/min). This is in good agreement with earlier findings [22,25,31].

### 3.2. SEM and EDS Analysis of Wet Etched Worn Inserts

**Figure 5:**
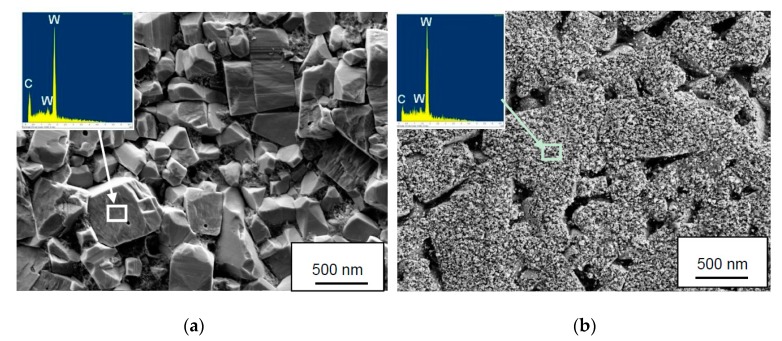
Surface characteristics of worn cemented carbide in the crater wear region as observed after removing the adhered work material by wet etching and energy dispersive X-ray spectroscopy (EDS) analysis obtained from the indicated areas. (**a**) Rough worn surface morphology due to attrition wear observed at low cutting speed and (**b**) smooth worn surface morphology due to diffusion wear observed at high cutting speed. Note the fine nanometer scale surface morphology of the surface in (**b**).

Figure 5 shows the worn cemented carbide in the crater wear region after removing the adhered work material by wet etching. At low cutting speed (see Figure 5a) the worn cemented carbide surface is relatively rough and individual cemented carbide grains can be distinguished. In contrast, at high cutting speed (see Figure 5b) the worn surface is relatively smooth and shows a very fine nanometer scale surface morphology not present at low cutting speed. EDS analysis using a low accelerating voltage (3 kV) show the presence of W and C but with a lower C-content than expected from WC stoichiometry. The differences in surface morphology are related to different active wear mechanisms, attrition wear dominating crater wear at low cutting speeds while diffusion wear dominating crater wear at high cutting speeds. The above wear mechanisms have earlier been observed by Dearnley and Grearson to be the two competitive wear mechanisms of cemented carbide in the machining of Ti6Al4V [31].

### 3.3. SEM and EDS Analysis of Polished Cross-Sections

**Figure 6:**
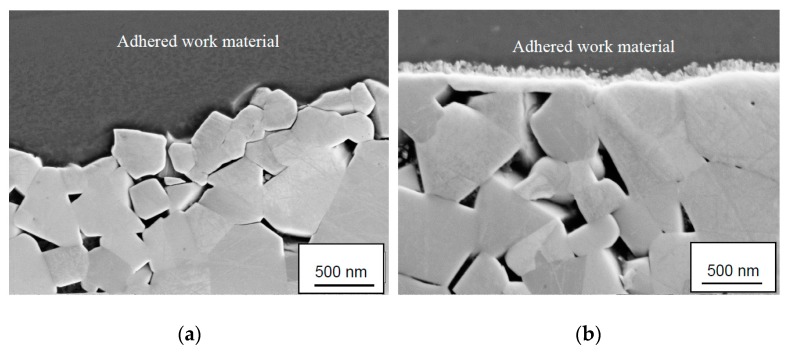
Cross-sections showing the worn cemented carbide in the center of the crater wear region. (**a**) Rough surface morphology due to attrition wear observed at low cutting speed and (**b**) smooth worn surface morphology due to diffusion wear observed at high cutting speed. Note the fine nanometer scale surface morphology of the diffusion layer in (**b**).

Figure 6 shows polished cross sections of the worn cemented carbide in the crater wear region illustrating the differences in wear characteristics observed at low and high cutting speed (see Figure 5). While the attrition wear mechanism results in a rough surface the diffusion wear mechanism results in a very smooth surface. As can be seen in Figure 6b, the diffusion wear mechanism is associated with the formation of a new phase with bright contrast at the interface (see the fine nanometer scale surface morphology in Figure 5b). The higher brightness of the formed phase, as compared with WC, indicates that it is formed due to carbon depletion of WC something which is confirmed by EDS-analysis which is published in detail in another work [30]. The formation of a carbon depleted WC layer at the adhered work material-cemented carbide interface is in good agreement with recent findings by Odelros et al., Kaplan et al. and Latteman et al. [26,27,28]. In contrast, the presence of scattered particles in the adhered work material layer, as observed by these authors, were not revealed in the present study.

### 3.4. AES Depth Profiling within the Crater on Worn Insert

**Figure 7:**
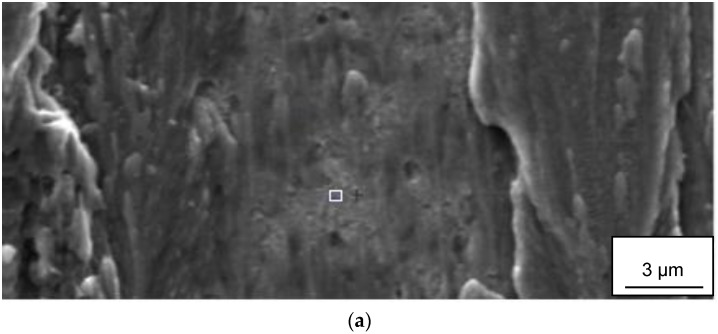

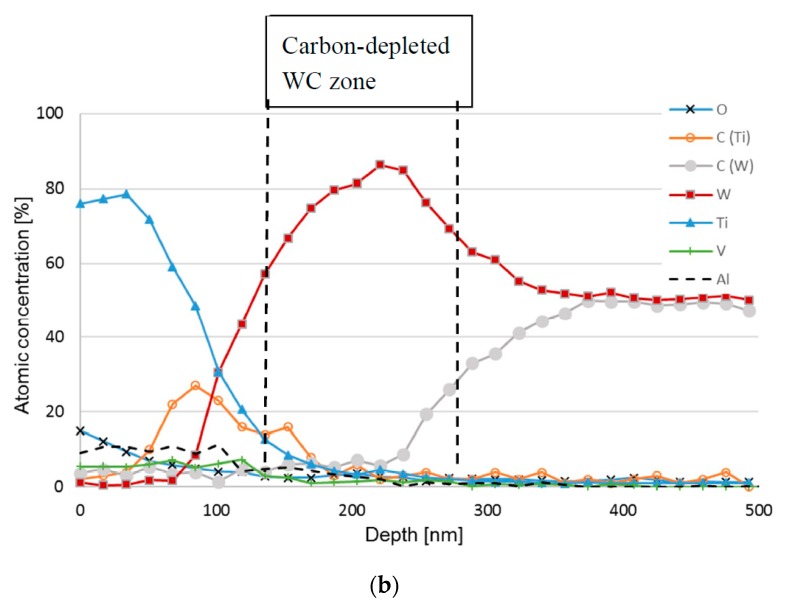
(**a**) Scanning electron microscopy (SEM) image of an area in the center of the worn crater (cutting speed: 90 m/min). (**b**) Auger electron spectroscopy (AES) depth profile obtained from the indicated area.

Figure 7 shows an AES depth profile obtained from the center of a crater formed on the rake face of a cemented carbide insert used for turning at high cutting speed. In order to limit the final sputter depth and improve the depth resolution of the depth profile an area with a thin adhered work material layer was chosen by finding an area where the layer appears almost transparent at 10 keV primary electron energy. This approach limits the layer thickness to 0.1–0.2 µm and reduces the tendency to preferential sputtering, ion beam induced roughness and atomic mixing effects.

**Figure 8:**
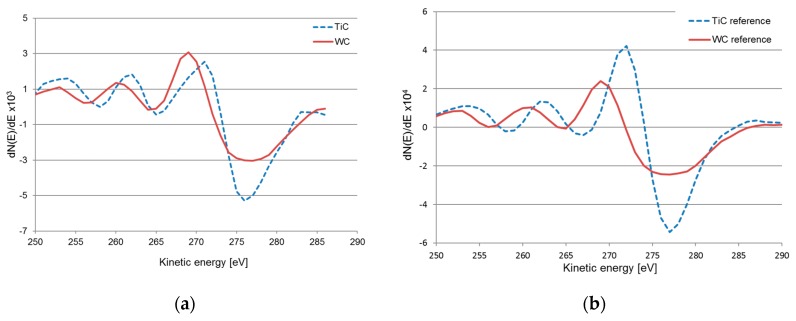
(**a**) Two different C KLL Auger spectra extracted from a carbon depth profile. C in WC (red spectra) and C in TiC (blue spectra), (**b**) carbon signals from reference samples of WC and TiC.

The AES depth profile clearly shows the presence of an approximately 150 nm thick carbon depleted layer in the WC phase (see Figure 6b). By using the linear least squares (LLS) routine two different components of the carbon depth profile can be distinguished, i.e., C in WC (C(W)) and C in TiC (C(Ti)) (see Figure 8a). Figure 8b shows the C KLL Auger spectra from reference samples of TiC and WC, respectively. While the main Auger transition, C KL_23_L_23_, of the two spectra is positioned around similar kinetic energy, i.e., 276 eV for WC and 277 eV for TiC, the main difference between the C KLL Auger spectra can be seen in the fine structure between 250 and 270 eV where the C KL_1_L_1_ and C KL_1_L_23_ transitions show different energy positions. For TiC these Auger transitions are at 258 eV and and 266 eV while for WC the transitions are at 256 eV and 264 eV, which is in good agreement with published data [32]. The formation of a carbon depleted layer WC layer and the presence of TiC at the interface indicates that degradation of WC is controlled by diffusion of carbon into the adhered work material. Although the AES results indicate the presence TiC at the adhered work material-cemented carbide interface, the presence of a continuous interfacial layer of TiC as proposed by Hartung and Kramer cannot be established [17].

### 3.5. AES Depth Profiling within the Crater on a Wet Etched Insert

**Figure 9:**
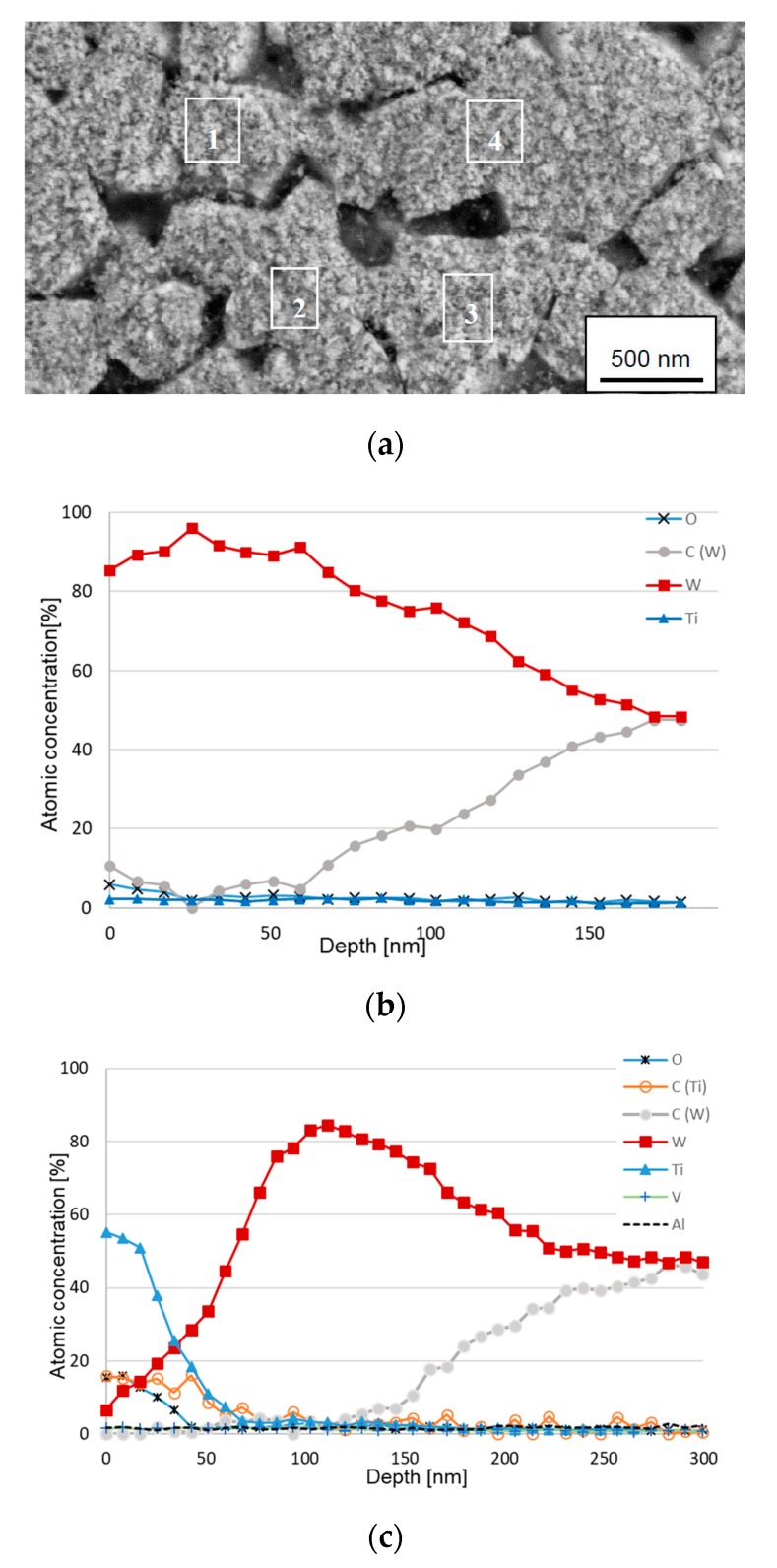
(**a**) SEM image of an area in the center of the worn crater after wet etching (cutting speed: 90 m/min). (**b**) AES depth profile obtained from areas 1, 2 and 3. (**c**) AES depth profile obtained from area 4.

**Figure 10:**
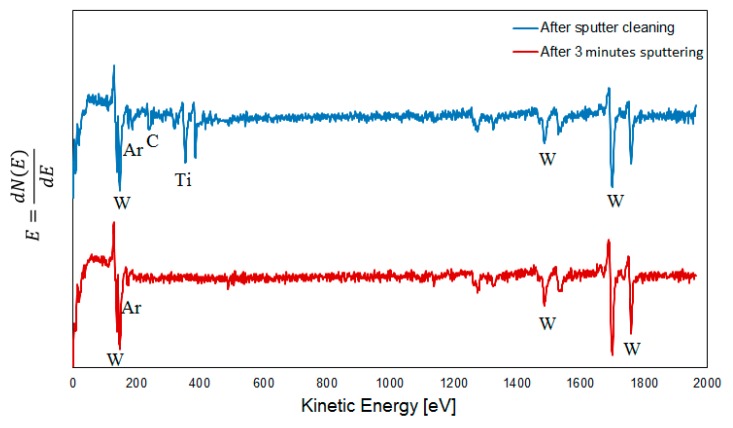
AES survey spectra obtained from area 4 after 5 s and 180 s sputtering. Note the presence of Ti (TiC) in the top surface.

Figure 9 shows AES depth profiles obtained from the center of a crater formed on the rake face of a cemented carbide insert used for turning at high cutting speed after wet etching. As can be seen the AES depth profiles give a clearer picture of the chemical interactions taking place at the cemented carbide surface. AES depth profiles obtained from areas 1, 2 and 3 show that the fine scale surface morphology in Figure 5b mainly corresponds to a pure W-phase with a thickness of 50–60 nm after which the W-signal decreases and the C-signal increases reaching the expected composition of WC at a depth of approximately 130 nm (in good agreement with the depth profile in Figure 7). However, one of the AES profiles, area 4, also reveals the presence of TiC in the surface, which suggests that C originating from the WC phase in the cemented carbide has diffused outwards and reacted with Ti forming TiC. This is also illustrated by the AES survey spectra in Figure 10. After 5 s sputtering, C, Ti and W are detected, while after 180 s sputtering only W is detected (the Ar signal in the two spectra originates from the Ar ion sputtering). Detailed analysis of the shifts in the C KLL spectrum after 5 s sputtering reveal that it corresponds to C in TiC.

### 3.6. AES Line Scan Analysis of Polished Cross-Sections

**Figure 11:**
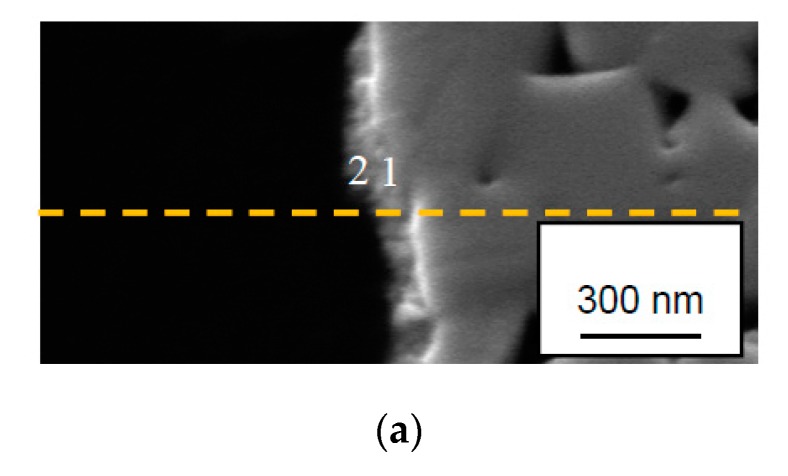

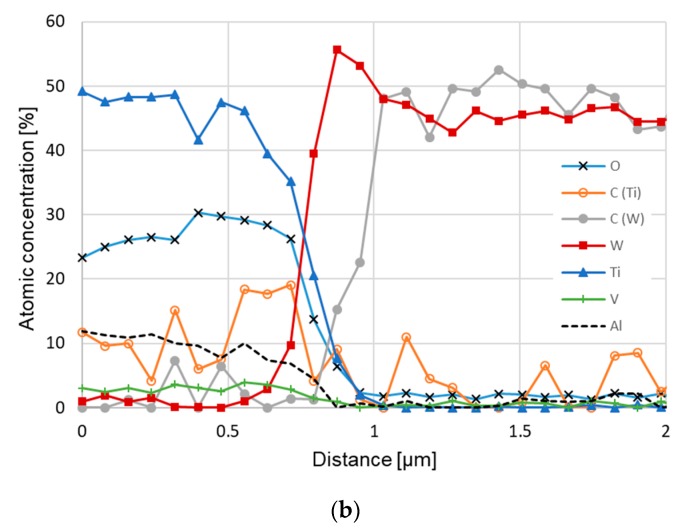
(**a**) Cross section of the rake face after 30 s turning Ti-6Al-4V at cutting speed of 90 m/min and feed rate of 0.2 mm/rev, (**b**) AES line scan obtained in the indicated line across the adhered work material–cemented carbide interface in the center of the crater wear region.

**Figure 12:**
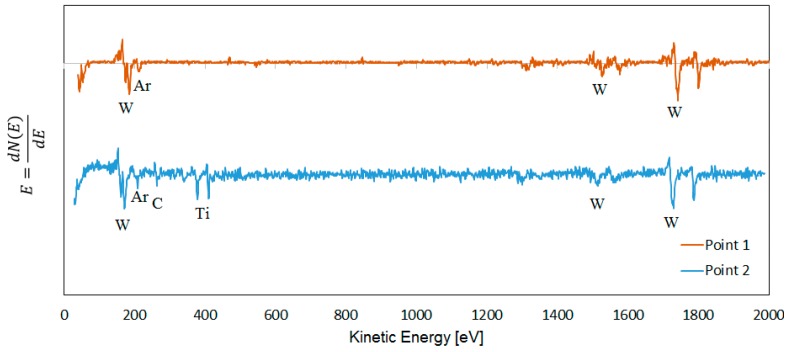
AES survey spectra obtained after 5 s sputtering in a point in the center of the diffusion layer (point 1) and in a point in the diffusion layer close to adhered work material (point 2). Note the presence of Ti (TiC) in point 2.

Figure 11 shows an AES line-scan across the cemented carbide–adhered work material interface in the center of a crater formed on the rake face of a cemented carbide insert used for turning at high cutting speed. The presence of a carbon depleted WC zone (the gap between the rise of the W signal and the C signal) is clearly revealed. The thickness of the carbon-depleted WC zone is around 150 nm, which is in good agreement with the results obtained from AES depth profiling (see Figure 7 and Figure 9). The line scan also reveals the presence of TiC in the interface towards the adhered work material (see Figure 7 and Figure 9). AES spot analysis in the outer part of the interfacial layer and in its center show the presence of TiC in the outer part, i.e., towards the adhered work material (see Figure 12). This is in good agreement with the results presented in Figure 10.

**Figure 13:**
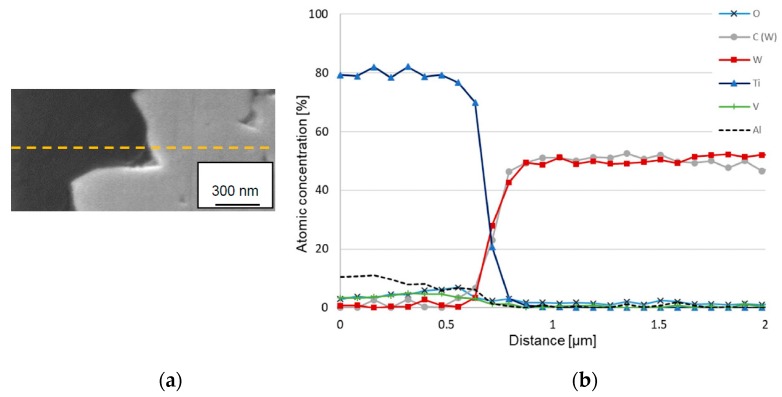
(**a**) Cross section of the rake face after 30 s turning Ti-6Al-4V at cutting speed of 30 m/min and feed rate of 0.2 mm/rev, (**b**) AES line scan obtained in the indicated line across the adhered work material–cemented carbide interface in the center of the crater wear region.

At the lower cutting speed (see Figure 13), the interface between the cemented carbide and adhered work material is very sharp and does not show any tendency to chemical degradation of the WC-phase.

### 3.7. Surface Analysis of Back-Side of Chips

**Figure 14:**
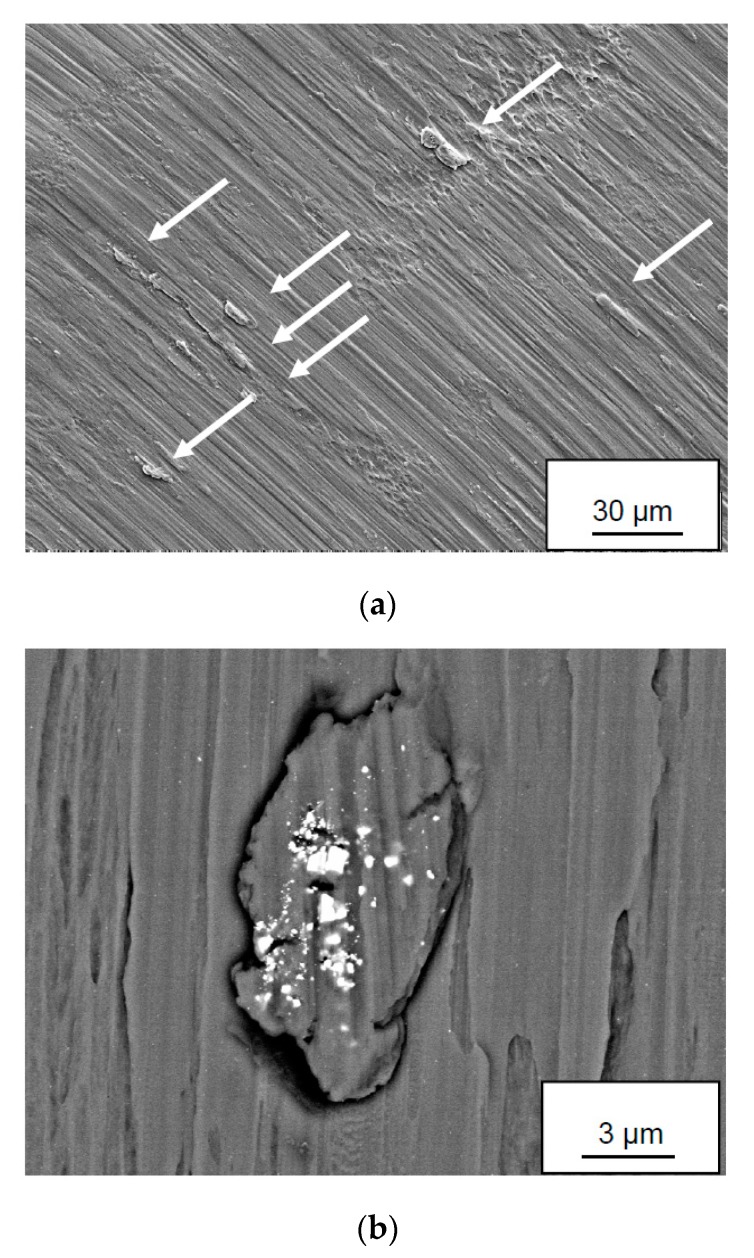

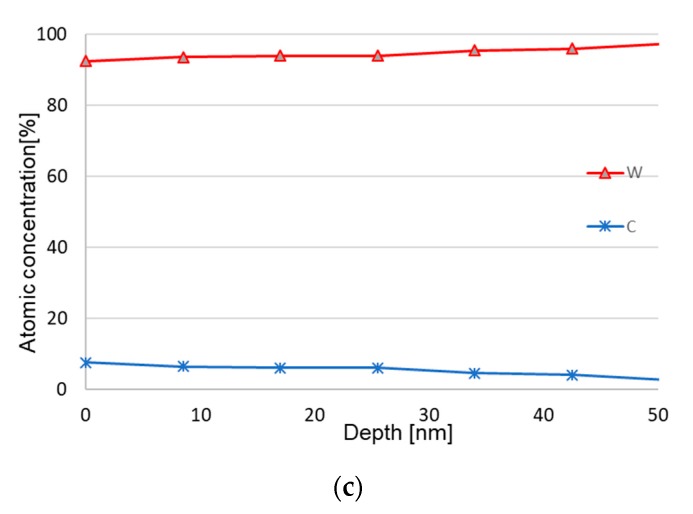
(**a**,**b**) SEM images of the backside, i.e., the surface in contact with the rake face, of a Ti-6Al-4V chip showing the presence of “islands” containing W-fragments (white fragments in Figure 13b) originating from the diffusion layer. (**c**) AES depth profile of a W-fragment.

**Figure 15:**
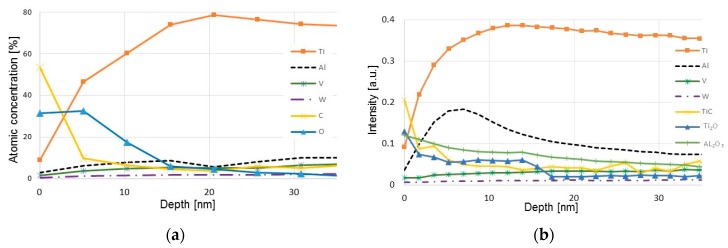
(**a**) AES and (**b**) time of flight secondary ion mass spectroscopy (ToF-SIMS) depth profiles of the backside—i.e., the surface in contact with the rake face—of a Ti-6Al-4V chip.

SEM of the back-side of the Ti6Al4V chips produced during turning at high cutting speeds revealed a relatively smooth surface with small “islands”, some 100 µm^2^ in size, protruding some micrometers from the chip surface (see Figure 14). In common for these “islands” is the presence of fine (sub-µm) wear particles originating from the cemented carbide. The high lateral resolution in combination with the high surface sensitivity of the AES-technique makes it possible to analyze the chemical composition of these wear fragments. The depth profile in Figure 14c shows that the analyzed fragment consists of W, i.e., that it originates from the diffusion layer formed at the cemented carbide–work material interface. It should be noted that AES analysis of areas outside the small “islands” (see Figure 15a) does not reveal any W or WC, i.e., the presence of W seems to be restricted to the “islands”.

In combination with the AES analysis, ToF-SIMS analysis (ToF-SIMS having a very high surface sensitivity and a trace element detection limit in the ppm-range) was used to analyze the diffusion of tungsten into the chip. The ToF-SIMS depth profiles in Figure 15b shows that the chip surface is covered with a thin (Ti,Al) oxide film with a thickness of around 15–20 nm. The oxide film is most probably formed during cooling of the chip from the cutting temperature to room temperature. More importantly, no atomic diffusion of W could be detected in the chip surface.

## 4. Discussion

In the present study, a methodology aimed at systematically analyzing the diffusion wear mechanisms of cemented carbide cutting tools in machining is proposed. It is shown that the steps taken and the experimental techniques used are sufficient to quantify thicknesses of element gradients in the materials in and near the contact zone after machining, which is relevant for modeling and verification of proposed wear mechanisms. Although much understanding of wear mechanisms can be gained from studies of independent systems such as diffusion couples, it is ultimately beneficial to provide data from actual cutting zones that can be compared in models for, e.g., growth or diffusion rates. The present work was based on turning experiments in Ti6Al4V and post-test characterization of the two tribo surfaces, i.e., the worn cemented carbide rake face surface and the back-side of the produced chips. For these materials, the previously suggested formation of a layer of TiC and a carbon-depleted region consisting mostly of W was confirmed, but thicknesses and element concentrations were also quantified for the first time in a cutting zone. No significant changes in these results were found when comparing different cutting times. The difference between this observation and the results from Olander and Heinrichs [16] could be due to the initial reaction stage that they observed already being worn off at our first measurements, meaning that the wear process was in equilibrium during our entire test series II. The basic steps in the methodology include:

(i) Characterization of the worn inserts using SEM and EDS followed by characterization of the worn inserts after wet etching when the adhered work material is removed and the worn cemented carbide is exposed. The above characterization is combined with AES depth profiling of the worn surfaces.

(ii) Characterization of polished cross-sections of worn as-tested inserts using SEM. The characterization is combined with AES line-scan analysis of the cemented carbide–work material interface.

(iii) SEM, AES and ToF-SIMS analysis of the back side of the produced chips, i.e., the surface in contact with the rake face of the cemented carbide insert.

A common problem when analyzing the wear mechanisms of cutting tools is the presence of adhered work material on the worn cutting tool surfaces. Somewhat surprisingly, rather few studies in the open literature have used wet etching to remove the work material and expose the worn cemented carbide for post-test surface characterization. Recent article by Kaplan et al. [27] establish decarburization of WC in contact with Ti-alloy both through machining experiments and diffusion couples using EDS technique to analyze etched worn insert. The EDS technique has poor lateral resolution, and performing AES analysis on an etched worn cutting tool provides clearer information which can be of use in understanding the wear mechanism. In the present study the use of wet etching followed by surface characterization was found to be a suitable and quick technique in order to understand the degradation and wear of cemented carbide in the turning of Ti6Al4V. Thus, the selection of an appropriate etching solution, temperature and time may significantly facilitate the experimental work. In combination with the preparation and characterization of polished cross-sections of worn as-tested inserts it offers a scientific approach to evaluate the surface degradation and wear mechanisms of cemented carbide in machining.

The present work also illuminates the potential of AES analysis when it comes to analyzing the degradation of cemented carbide in contact with Ti6Al4V during turning. The high surface sensitivity in combination with high lateral resolution make it possible to analyze the worn cemented carbide surface on a sub-µm level. Especially AES sputter depth profiling, resulting in detailed information of variations in chemical composition across interfaces, is a powerful tool when it comes to understanding diffusion wear.

Also, the present work illustrates the potential gain from analyzing not only the worn tool but also the produced chips. An accurate characterization of the back-side of the chips will give important information regarding the wear mechanisms taking place at the tool rake face–chip interface. Surface analysis techniques such as AES and ToF-SIMS are well suited for this type of surface characterization.

## 5. Conclusions

- The results show that the characterization of wet etched worn inserts give quick and useful information regarding the diffusion degradation of cemented carbide, in the present work the formation of a fine crystalline W layer (carbon depleted WC layer) at the tool–chip interface.
- AES-depth profiling of degradation layers gives detailed information of the layer thickness and the formation of secondary phases; in the present study TiC is formed as revealed by the shift in the carbon-signal.
- In contrast, characterization of as-tested worn inserts is more complicated, since the worn tool regions are generally covered by adhered work material. AES-depth profiling through these layers may give some complementary information, but large analyzed areas and thick adhered layers should be avoided to obtain well defined depth profiles.
- AES-line scan analysis of cross sections is an interesting valuable approach which may also give complementary information.
- Surface characterization of the back-side of the produced chips will increase the understanding regarding the actual tool wear process, i.e., the removal of wear fragments from the cemented carbide insert. In the present study, fine W particles were found on the back-side of the chip.

Using SEM, AES and ToF-SIMS, it was confirmed that wear of the cemented carbide is mainly caused by the dissolution of WC at the tool-chip interface forming a W layer which subsequently is removed by the chip flowing over the tool rake face.

## Figures and Tables

**Table 1:** Hardness and chemical composition of the tool grade according to the material specification provided by AB Sandvik Coromant.

WC [vol%]	Co [vol%]	WC Grain Size [µm]	Hardness, HV_3_
89.8	10.2	1	1580

**Table 2:** Hardness and chemical composition (weight %) of the Ti6Al4V according to according to the material specification provided by material supplier Timet.

Al	V	Fe	C	O	N	Y	Ti	Hardness, HV_5_
6.425	3.970	0.155	0.019	0.190	0.006	<0.001	Bal.	300
